# A Socioecological Approach to Understanding Why Teachers Feel Unsafe at School

**DOI:** 10.3390/bs15091149

**Published:** 2025-08-23

**Authors:** Verónica López, Luis González, Rami Benbenishty, Ron Avi Astor, Javier Torres-Vallejos, Tabata Contreras-Villalobos, Juan San Martin

**Affiliations:** 1 School of Psychology, Pontificia Universidad Católica de Valparaíso, Viña del Mar 2530388, Chile; 2 Centro de Investigación para la Educación Inclusiva, Pontificia Universidad Católica de Valparaíso, Viña del Mar 2530388, Chile; luis.gonzalez.h@pucv.cl; 3 School of Social Work and Social Welfare, Hebrew University of Jerusalem, Jerusalem 91905, Israel; ramibenben@gmail.com; 4 Faculty of Social Sciences and Education, Universidad Andrés Bello, Santiago 8370035, Chile; 5 Department of Social Welfare, Luskin School of Public Affairs, University of California, Los Angeles, CA 90095, USA; astor@luskin.ucla.edu; 6 School of Education and Information Studies, University of California, Los Angeles, CA 90095, USA; 7 Faculty of Social Sciences, School of Psychology, Universidad Santo Tomas, Santiago 8370003, Chile; jtorresvallejos@santotomas.cl (J.T.-V.); juansanmartinve@santotomas.cl (J.S.M.); 8 Department of Educational Organization, Faculty of Education, Universidad de Barcelona, 08035 Barcelona, Spain; tabata.contreras@ub.edu

**Keywords:** school violence, victimization, teachers, school climate, socioecological perspective

## Abstract

Despite the increased research on violence toward teachers and public policies aimed at protecting teachers from violence, knowledge of the factors contributing to teachers’ sense of safety at school remains limited. Drawing from socioecological theory, we examined the contributions of both teachers’, parents’, students’, and schools’ characteristics to teachers’ sense of feeling unsafe in school. Specifically, we examined teachers’ individual and work characteristics (sex, age, years of experience, and working in the regular classroom or not), their perceptions of school violence, and their relationships with students and their peers. At the school level, we examined the school size, poverty level, and school-level reports of parents’, students’, and teachers’ perception of the school climate and school violence. The sample consisted of 9625 teachers (73% female), 126,301 students, and 56,196 parents from 2116 schools with a low socioeconomic status in Chile. Descriptive statistics showed that most teachers do not feel afraid (72.9%) nor thought that their job was dangerous (74.6%). A hierarchical multivariate regression analysis and multilevel analyses showed that teachers with higher perceptions of feeling unsafe were females or reported being “other sex”, had fewer years of experience, worked mainly in the classroom, perceived a higher level of school violence, and had worse perceptions of peer relationships and teacher–student relationships. These teachers were mostly in schools with higher poverty levels, larger enrollment, and higher student-reported and parent-reported school violence compared to the rest of the sample of low-SES Chilean schools. We discuss the implications of these findings for preventive school interventions and programs regarding school violence and teacher turnover.

## 1. Introduction

In recent years, an increasing amount of research has drawn attention to the fact that teachers are not passive observers of school violence but are also affected by different forms of victimization (Astor et al., 2024; Benbenishty et al., 2019; Dirzyte et al., 2024; Martinez et al., 2025; McMahon et al., 2024a, 2024b; Perry et al., 2024; Reddy et al., 2023; Yang et al., 2022). A meta-analysis reports that the prevalence of any teacher-reported violence victimization within two years ranged from 20% to 75% with a pooled prevalence of 53% (Longobardi et al., 2019). Teachers’ victimization has many negative outcomes, including mental health (McMahon et al., 2024a, 2024b), well-being (Dirzyte et al., 2024; Reddy et al., 2023; Yang et al., 2022), job satisfaction, and retention (McMahon et al., 2024b; Perry et al., 2024).

Given its prevalence and consequences, there is an increased interest in preventing teachers’ victimization. In countries such as Mexico, Colombia, and Chile, teacher unions have argued that teachers are starting to feel unsafe in their schools, indicating that an increase in medical leave and teacher turnover might be associated with violence toward teachers (López et al., 2020; Márquez et al., 2022; Rocha, 2021). In some countries, teacher unions and education departments have advocated different ways to protect teachers’ safety and emotional well-being. One of these ways is through legal regulations ensuring teacher safety (Mokonyane et al., 2024). In Chile, a bill promoting a positive school climate and protecting teachers against school violence is currently being discussed in the Senate (Senado República de Chile, 2025).

There is an increased interest in research on violence toward teachers (Robinson et al., 2025). However, the interplay between individual and contextual factors that may explain why teachers feel unsafe in their schools is not yet fully understood. In this study, we sought to answer the following research question: What individual and school characteristics contribute to teachers’ sense of safety at school?

### 1.1. School Violence from a Socioecological Perspective

The aim of the present study is to examine factors that can explain teachers’ perceptions of feeling unsafe in school. We base our study to a large extent on the “School Violence in Context” theory (Astor & Benbenishty, 2019). This socioecological theory suggests that teachers’ perceptions of safety are influenced by their individual characteristics and experiences, as well as their interactions with the school as a context. The school context encompasses the broader environments in which the school is situated (e.g., poverty) and the personal experiences, perceptions, and interactions among multiple school actors, including students, other teachers, and parents.

Research on violence toward teachers has shown that it affects them both emotionally and professionally (Anderman et al., 2018). In countries such as Mexico, Colombia, and Chile, teacher unions have argued that teachers are starting to feel unsafe in their schools, indicating that an increase in medical leave and teacher turnover might be associated with violence toward teachers (López et al., 2020; Márquez et al., 2022; Rocha, 2021). In some countries, teacher unions and education departments have advocated different ways to protect teachers’ labor rights and emotional well-being. For example, sufficient legal measures are required to regulate teacher safety, ensuring their protection in a robust regulatory framework (Mokonyane et al., 2024). In Chile, a bill on school climate that protects teachers against school violence is currently being discussed in the Senate (Senado República de Chile, 2025).

However, an emerging body of research has also provided evidence that teachers may also, intentionally or not, participate in different forms of school violence. For example, López et al. (2020) showed a positive association between students victimizing teachers and reporting being victimized by their teachers. Although there are cultural patterns involved in these forms of relational victimization between teachers and students (López et al., 2020), comparative studies have shown a persistent association between teachers being victimized by and victimizing students (Benbenishty et al., 2019).

From a socioecological perspective (Astor & Benbenishty, 2019), school violence is embedded in social, relational, and interpersonal contexts that are interdependent. Teachers’ sense of insecurity at school might also be attributed to other relationally aggressive patterns of interaction, such as hostile verbal interactions between parents and teachers, among teachers, and between teachers and school leaders. Noncommunicative and hostile environments might lead to unintended difficulties in resolving conflicts between members of the school community through dialogue. An interactional and relational pattern of behavior that becomes persistent over time might lead to violent acts, that increase teachers’ sense of feeling unsafe at school. However, to date, these other relational forms of school violence as perceived by multiple actors have gone understudied.

### 1.2. Individual Factors Contributing to Teachers’ Sense of Safety at School

Vulnerability theories of fear of victimization in school (e.g., Swartz et al., 2011) suggest that there are individual factors that influence teachers’ fear of victimization. One such factor is the teacher’s sex. The extant literature on fear of crime found that females tend to fear crime more (Öztürk et al., 2016). However, international findings regarding the effects of teachers’ gender in schools are less conclusive. A study by De Cordova et al. (2019) with an Italian sample found that female teachers reported higher levels of violence perpetrated by parents, lower support from the administrative team, and lower satisfaction in their relationships with both parents and students. In contrast, Ghadban et al. (2023), in a study of Israeli teachers, found no significant relationship between gender and victimization.

Some studies have focused on the influence of professional teaching experience, a variable associated with age, on outcomes related to perceptions of safety. According to a qualitative study by Berkowitz et al. (2022), teachers reported that having less experience hinders the establishment of boundaries and proper classroom management, promoting disrespectful behavior among students and exposing teachers to violence. A quantitative study found that teachers with more years of service reported a lower risk of violence perpetrated by students compared to teachers with fewer years of experience (De Cordova et al., 2019). The effect of years of teaching experience on teachers’ sense of safety was also evidenced in many studies authored by the APA Task Force on Violence Against Educators and School Personnel (Astor et al., 2024; McMahon et al., 2024a, 2024b; Watson et al., 2024).

Teachers’ sex may influence their vulnerability to victimization. However, international findings regarding gender effects are less conclusive. A study by De Cordova et al. (2019) with an Italian sample found that female teachers reported higher levels of violence perpetrated by parents, lower support from the administrative team, and lower satisfaction in their relationships with both parents and students, compared to male teachers. In contrast, Ghadban et al. (2023), in a study of Israeli teachers, found no significant relationship between gender and victimization.

Additionally, as teachers may have different responsibilities in school, their role may influence their vulnerability to victimization and feelings of safety. One would expect, for example, that teachers who have management roles may be less vulnerable compared with teachers in class who interact with students daily. However, we did not find a study that addressed the issue of the teacher’s role in school.

### 1.3. School Factors Contributing to Teachers’ Sense of Safety at School

Teachers’ sense of feeling unsafe in schools is influenced by various factors, ranging from physical safety concerns to psychological and systemic pressures (Astor et al., 2024; Martinez et al., 2025). These factors can significantly affect their mental health (McMahon et al., 2024a, 2024b), well-being (Dirzyte et al., 2024; Reddy et al., 2023; Yang et al., 2022), job satisfaction, and retention (McMahon et al., 2024b; Perry et al., 2024). Certain types of school contexts might play a relevant role in how teachers experience feeling unsafe at school. School characteristics such as the school’s socioeconomic status (SES) and school size (i.e., student enrollment) may contribute, because these meso-level characteristics shape the complexity of the school in which teachers work. In most countries, low-SES schools tend to be in low-SES neighborhoods, which might contribute to teachers’ reports of feeling unsafe; there is evidence of this association in Chile for students (López et al., 2017) but not for teachers. Teacher-perceived safety might also be influenced by school size, because a small school might contribute to feelings of control and self-efficacy, making teachers feel more “at home” than in larger school settings. In a study on the evolution of indicators of school violence and school climate in Chile, López et al. (2025b) found that students in smaller schools—i.e., those with fewer than 200 students—perceived a more positive school climate and reported lower peer victimization than schools with more than 800 students. From a socioecological perspective, contexts shape interactions (Astor & Benbenishty, 2019; Berkowitz et al., 2022; Claussen et al., 2022) and, therefore, the physical environment and potentially closer interactions in smaller schools might enable teachers to engage in more positive day-to-day interactions with their students, which the literature recognizes as a key element for students’ school engagement (April et al., 2023; Cerveny et al., 2022).

In this study, we argue that teachers’ sense of safety is also influenced by the presence of hostile, violent, or noncommunicative relationships among different members of the school community based on their experiences and perceptions. Some studies highlighted how episodes related to verbal threats, physical assaults, and property damage by students contributed to teachers feeling unsafe (Reddy et al., 2023). Other studies focused on positive organizational and systemic factors such as the school climate, which can also play a significant role. Teachers’ perceptions of negative relational patterns in the school community (i.e., violent, uncommunicative, or hostile climates) also influence their sense of safety overall (Astor et al., 2024). Conversely, a supportive school climate characterized by positive relationships with colleagues (i.e., collegial relationships) and visible, supportive leadership enhances teachers’ psychological safety (Bagga, 2022; Capp et al., 2020, 2021, 2022). As Capp et al. (2022) argued, school climate is not a singular experience but an interplay of various elements that include leadership support, collegial relationships, organizational structure, and shared beliefs among staff members. Positive school climates are marked by supportive leadership, a sense of collaboration, and organizational practices that foster respect and inclusion. Conversely, poor climate conditions include feelings of isolation, distrust in the administration, and fragmented communication. Therefore, negative school climates and lack of administrative support can increase feelings of vulnerability (Bagga, 2022). Likewise, conflicts with parents and other members of the community can also affect teachers’ sense of safety. These interactions often stem from differing views on education and can lead to additional stress and pressure on teachers (Chorna et al., 2022; Dębowski & Lubicz Miszewski, 2024). Hence, in this study, we posited that teachers’ sense of safety is associated with not only violence by students but also with noncommunicative, conflictive relationships and violent relationships among members of the school community, which create a hostile and perhaps violent school environment. These school factors have rarely been studied together. Furthermore, teachers’ individual characteristics such as age and sex and other school contexts such as SES and school size have not been examined in conjunction with relational climate factors regarding safety. Therefore, this study examined the joint contributions of student-reported, parent-reported, and teacher-reported measures of school violence and school climate on Chilean teachers’ perception of safety at school in the contexts of their individual characteristics (age and sex) and their school’s characteristics (SES and size).

## 2. Materials and Methods

### 2.1. Sample and Participants

The present study is a secondary analysis of data originally collected by the National Board of Assistance and School Scholarships (abbreviated JUNAEB in Spanish) through its *Programa Habilidades para la Vida* (Skills for Life Program; hereafter, SFL), a public-school mental health program run by the JUNAEB, which includes this as part of their school climate and school violence monitoring system for students enrolled in upper elementary grades, which, in Chile, correspond to fifth, sixth, seventh, and eight grade. The sample consisted of 9639 teachers (72.6% female) from 2116 low-SES elementary schools in Chile. All participating schools were part of the SFL program. SFL is a focalized program catered to the country’s lowest-SES schools, meaning that schools are eligible to participate in the program based on their low SES, which is measured as the percentage of high-risk students enrolled in the school, adjusted by school size. In Chile, these lower-SES schools usually cater to a greater proportion of students with disabilities, migrant students, and students with challenging family situations.

The SFL school climate and school violence monitoring system surveys students from fifth to eighth grade, their teachers, and their parents, asking about their perceptions of school climate and school violence. In this study, we used the teachers’ responses as the initial dataset and, later, merged the aggregated responses of students and parents using each school’s unique ID. Therefore, this was a multi-informant study.

The total number of teachers who answered the questionnaires was 11,608; and 9639 (response rate = 83.0%) had full information for the variables of interest and were included in this study. Teachers were from 2116 schools, and we aggregated the responses of students (*N* = 126,301) and parents (*N* = 56,196) for each school.

### 2.2. Dependent Variable: Feeling Unsafe at School

Teachers’ perception of feeling unsafe was measured using two self-reported items: “I feel afraid of being attacked at school” and “I think that my job is dangerous.” Both items had a 4-point Likert scale (0 = strongly disagree to 3 = strongly agree). The correlation between these variables was *r* = 0.68. These variables were averaged to create the measure of feeling unsafe at school.

### 2.3. Independent Variables

#### 2.3.1. Characterization of Teachers and Their Work

Sex. Teachers reported their sex (0 = female, 1 = male, 2 = other).

Age. Teachers reported their age, given prespecified categories (0 = 18–30, 1 = 31–45, 2 = 46 or older).

Years of Experience. Teachers reported how many years they had worked as a teacher.

Main Role. This dichotomous variable indicated whether the teacher’s main role in the school is primarily outside of the classroom (e.g., inspector, school climate manager; 0 = primarily inside the classroom, 1 = primarily outside the classroom).

#### 2.3.2. Teachers’ Perceptions of School Climate and School Violence

Positive Collegial Relationships. This variable was measured using one item: “I think I have a good relationship with my colleagues.” Responses were measured on a scale from 1 (strongly disagree) to 4 (strongly agree) (Benbenishty & Astor, 2005). We calculated the school mean of the index to create an aggregated value.

Positive Teacher–Student Relationships. This variable was measured using two items addressed by teachers: “I think there is a good relationship between teacher and students” and “In this school, teachers support their students.” The correlation between these questions was *r* = 0.62. Responses were measured on a scale from 1 (strongly disagree) to 4 (strongly agree) (Benbenishty & Astor, 2005). We generated an index by averaging the responses and calculated the school mean of the index to create an aggregated value.

Perceived School Violence. Teachers responded to the question “What is the magnitude of the problem of school violence at your school?” Responses were measured on a scale of 1 (is not a problem) to 5 (is a really big problem) (Benbenishty & Astor, 2005). We calculated the school mean of the item to create an aggregated value.

#### 2.3.3. School Characteristics

School Poverty Index. This measure is known in the Chilean school system as the Index of School Vulnerability (*Indice de Vulnerabilidad Escolar*, IVE), is computed by JUNAEB, and reflects the percentage of vulnerable students enrolled in the school. JUNAEB defines vulnerability (i.e., poverty) as a dynamic condition resulting from the interaction of individual and contextual risk and protective factors (e.g., family, school, and community), which influence children’s biopsychosocial, cultural, and environmental development and well-being. Since 2006, student vulnerability has been assessed using the IVE-SINAE index, primarily based on socioeconomic status, academic performance, and school dropout risk. Recently, JUNAEB adopted a multidimensional approach to better capture vulnerability across the educational trajectory, progressively integrating this revised measure into the criterion for targeting and allocating student supports (JUNAEB, 2025).

School Size. This variable represented the total number of students enrolled in the school.

#### 2.3.4. Students’ and Parents’ Perceptions of School Climate and School Violence

Student-Reported Peer Victimization. We used the School Victimization Scale developed by Furlong (1996), modified for use in Israel (Benbenishty & Astor, 2005), and later adapted to fit the Chilean context (López et al., 2014). It features 27 items that ask students about the frequency of violent episodes involving classmates at the school during the previous month (0 = never, 1 = once or twice, 2 = three or more times). A sample item of this measure is “A student humiliated you or made you feel bad”. It evaluates physical, verbal, social, cyber, and sexual dimensions of victimization (López et al., 2014). We generated an index by averaging the responses (Cronbach’s α = 0.96). Then, we calculated the school mean of the index to create an aggregated value.

Student-Reported Physical Victimization of Teachers. Six items assessed self-reports of students regarding victimizing their teachers during the last month (0 = not at all, 1 = once or twice, 2 = three or more times). A sample item of this measure is “You kicked or slapped a teacher”. These questions were taken from López et al. (2020). We computed an index on the student level using the average of all items (Cronbach’s α = 0.85). Then, we calculated the school mean of the index to create an aggregated value.

Student-Reported Victimization by Teachers. Three items were used to describe victimization of students by teachers as reported by students, using the stem “Last month, a teacher…” with the following behaviors: “mocked, insulted, or humiliated you,” “seized and shoved you on purpose,” and “pinched you or slapped you” (0 = not at all, 1 = once or twice, 2 = three or more times). These questions were taken from Astor and Benbenishty (2019) and have been used in other studies (e.g., Arënliu et al., 2020). We computed an index on the student level using the average of all items (Cronbach’s α = 0.70). Then, we calculated the school mean of the index to create an aggregated value.

Student-Reported Social Support from Teachers. Three items were used to describe teacher support as reported by students: “If I need it, I can talk to a teacher at my school,” “My relationship with my teachers is good and close,” and “The teachers at my school care about their students” (0 = not at all, 1 = once or twice, 2 = three or more times). These questions were taken from Astor and Benbenishty (2019). We computed an index on the student level using the average of all items (Cronbach’s α = 0.73). Then, we calculated the school mean of the index to create an aggregated value.

Parent-Reported Victimization by Teachers. Two items assessed self-report of parents regarding being victimized by teachers during the last month: “A teacher humiliated me” and “A teacher physically attacked me”. The items were measured on a 4-point scale (0 = not at all, 1 = once, 2 = two or three times, 3 = four or more times) (Benbenishty & Astor, 2005). We computed an index on the student level using the average of all items (Cronbach’s α = 0.85). Then, we calculated the school mean of the index to create an aggregated value.

Parent-Reported Positive Relationship with School. This variable was measured using five items that assessed parents’ report about communication with and participation in the school. The items were as follows: “There is open communication between teachers and parents,” “They consider the parents’ opinion on the education of their children,” “The parents are informed about the children’s problem,” “The head teacher interviews the parents of his/her class at least once a year,” and “Parents participate by giving their opinions on important school decisions.” Responses were measured on a scale from 1 (strongly disagree) to 4 (strongly agree) (Benbenishty & Astor, 2005). We computed an index on the student level using the average of all items (Cronbach’s α = 0.79). Then, we calculated the school mean of the index to create an aggregated value.

### 2.4. Analytic Plan

We first calculated descriptive statistics of the study variables. Although the response rate of “other” regarding sex had a low frequency (0.86%), this percentage coincides with the latest national census of Chile (Ministry of Social Development and Family, 2023) which reported that 1.3% of the population identified as transgender or another gender. Therefore, we decided to retain these responses and not treat them as missing. We calculated Pearson’s correlations between the sense of feeling unsafe index and the continuous independent variables, along with analysis of variance between the sense of feeling unsafe and the categorical and dichotomous independent variables at the individual level. To test the relative contribution of a group of predictors to perceptions of feeling unsafe at school, we performed a two-level multilevel linear regression analysis, considering individual and school-level factors. Level 1 variables included teachers’ sex, years of experience, main job role, perceived violence at the school, and perceptions of positive collegial and teacher–student relationships. Level 2 variables included the schools’ SES and enrollment; school means of students’ perceptions of peer victimization, student-to-teacher victimization, teacher-to-student victimization, and teachers’ support; and school means of parents’ perceptions of teacher-to-parent victimization and school–parent relationship. Five multilevel models were fitted to examine associations with teachers’ feeling unsafe at school. A null model was estimated. Model 1 analyzed the effects of school characteristics (e.g., size and vulnerability). Model 2 included teachers’ characteristics (e.g., sex, experience, and main role). Model 3 included all school-level climate and victimization reports. Model 4 included the individual perceptions of teachers on school climate and violence. All analyses were conducted using Stata 15.

### 2.5. Ethical Considerations

Institutional review board approval was obtained from the first author’s institution. Participation was authorized and supported by the schools as part of a system of monitoring school climate and school violence among students in fifth to eighth grades. Teachers signed an informed consent form, and all participating schools received an informed consent statement guaranteeing voluntary participation, confidentiality of all information, and safeguarding of the students’ health and physical and psychological well-being. Additionally, students signed a similar informed assent for the study. Responses of teachers, parents, and students were anonymous. The deidentified database was provided by SFL to the first author, with a prior written memo of understanding that guaranteed confidentiality and ethical use of the information.

## 3. Results

Table 1 shows the means and standard deviations or percentages of the study variables. The sample primarily featured female teachers (72.5%), with a small proportion of teachers who responded “other” regarding sex (0.8%). Many teachers fell in the 31–45 age group (53.7%). Teachers had a mean of 13.51 years of experience (SD = 9.82), and 20.8% reported their main role occurred outside of the classroom. The mean of the feeling unsafe index was 0.97 (SD = 0.85), positive collegial relationships was 2.63 (SD = 0.58), and teacher–student relationships was 2.51 (SD = 0.52). Schools had a high mean for the vulnerability index (M = 0.87, SD = 0.09) and a mean enrollment of 346.44 students (SD = 237.02). Regarding the dependent variable of feeling unsafe at schools, results showed that 27.1% of teachers agreed or strongly agreed with being afraid of being attacked at school, and that 25.4% agreed or strongly agreed that their job was dangerous.

Bivariate associations between individual independent variables and feeling unsafe were calculated. Results from the analysis of variance indicated that feeling unsafe was not associated significantly with sex, *F*(2, 9636) = 24.21, *p* = 0.04, or age, *F*(2, 9636) = 8.68, *p* = 0.10). It was positively associated with the perceived severity of school violence (*r* = 0.43, *p* < 0.001) and negatively associated with years of experience (*r* = −0.07, *p* < 0.001), positive collegial relationships (*r* = −0.17, *p* < 0.001), and positive teacher–student relationships (*r* = −0.20, *p* < 0.001).

The results of the multilevel linear regression analysis of the teachers’ feeling unsafe at school are shown in Table 2. Model 1 showed that higher vulnerability at school was associated with a higher level of feeling unsafe (*b* = 1.154, *p* < 0.001), as was larger school enrollment (*b* = 0.001, *p* < 0.001).

Model 2 included variables related to teachers’ characteristics. In this model, the previously included variables did not change in directionality nor their level of significance. Male teachers were less likely to report feeling unsafe compared to female teachers (*b* = −0.048, *p* = 0.010), and females were less likely to report feeling unsafe compared to teachers who reported “other sex” (*b* = 0.439, *p* < 0.001). More years of experience (*b* = −0.005, *p* < 0.001) and working primarily outside the classroom (*b* = −0.171, *p* < 0.001) were also associated with fewer reports of feeling unsafe.

Model 3 added the school-level teachers’, students’ and parents’ perceptions of school climate and victimization. As in the previous model, the variables in Model 2 did not change their directionality and did not lose their statistical significance. The school means of teachers’ reports of positive collegial relationships (*b* = −0.186, *p* < 0.001) and positive teacher–student relationships (*b* = −0.238, *p* < 0.001) were negatively associated with feeling unsafe, where a higher school mean was linked to a lower index of feeling unsafe. Students’ and parents’ perceptions of school climate and victimization were not associated with feeling unsafe at school.

The final model included teachers’ perceptions of the school climate and school violence. In this full model, all variables maintained their coefficient and statistical significance except for the school means of teachers’ perceptions of a positive collegial relationship and positive teacher–student relationships, which decreased and became nonsignificant. Higher individual teachers’ perceptions of good collegial relationships were associated with lower indices of feeling unsafe (*b* = −0.104, *p* < 0.001), as did individual perceptions of good teacher–student relationships (*b* = −0.104, *p* < 0.001). A higher individual teachers’ perception of the severity of school violence was associated with a higher index of feeling unsafe (*b* = 0.275, *p* < 0.001).

The model fit improved progressively across the five estimated multilevel models. Adding predictors in subsequent models led to substantial improvements in the model fit, as reflected in the decreasing AIC and BIC values. The best-fitting model, Model 4, achieved the lowest AIC (21,734.01) and BIC (21,884.66), with a log-likelihood of –10,859.24. Using this criterion, these results indicate that the last model, including all the predictors, was the most parsimonious and well-fitted model.

The bottom part of Table 2 reports the variance in the dependent variable at the teacher and school levels and the explained variance compared to the null model. Considering these estimates, the differences in the perceptions of feeling unsafe at school were concentrated at the individual level.

Regarding the proportion of explained variance in the models, a higher proportion of individual and school-level variance was explained by variables associated with teachers’ perception of school climate and perceived violence (Model 4). Finally, in Model 4, the total proportion of the explained variance was 13.6% at the individual level and 60.2% at the school level.

## 4. Discussion

This study examined teachers’ feeling unsafe at school in the context of low-SES schools in Chile. Findings show that more than 70% of teachers disagreed or strongly disagreed with the assertions that they were afraid of being attacked in schools, and that they felt that their job was dangerous. In other words, most of the teachers do feel safe at school. The perception of feeling unsafe at school is mostly related to teachers’ individual characteristics and social identity markers. On one side, teachers who identified as female or a sex other than male or female were associated with a higher sense of feeling unsafe compared to male teachers. The results regarding female teachers having a higher perception of feeling unsafe is consistent with Astor et al.’s (2024) study in the United States and previous results in the Chilean context, in which young female teachers had higher emotional distress compared to other groups (López et al., 2020). They also coincide with De Cordova et al.’s (2019) study, which found higher victimization among female teachers in Italy compared to male teachers. Our findings contribute to the literature by providing initial evidence of higher levels of feeling unsafe at school among teachers who reported identifying as “other” regarding their sex (i.e., neither female nor male). To our knowledge, this is the first study to examine factors related to victimization among teachers who might possibly identify themselves as gay/lesbian, bisexual, transgender, or gender-nonconforming teachers in Chile. Societal norms in Chile still make it hard for teachers to publicly identify themselves as diverse in terms of their sexual orientation and/or gender identity; in this sense, the below one percent (<1%) of teachers in this study who expressed their sex as “other” (*N* = 83) is similar to the 1.7% of adults who in the last census identified themselves as gay/lesbian; 1.5% as bisexual; and less than 1% as transgender or having a gender identity different than their declared sex (Ministry of Social Development and Family, 2023). In this sense, the higher sense of feeling unsafe in this group suggests that schools can be a hostile environment for this group of teachers, and call on the need for schools and legislators to advocate and contribute to creating safer environments, not only for students but for the whole school community.

Consistent with research authored by the APA Task Force on Violence Against Educators and School Personnel (Astor et al., 2024; McMahon et al., 2024a, 2024b; Watson et al., 2024), the findings of this study show that teachers with fewer years of experience had higher reports of feeling unsafe. As Berkowitz et al. (2022) posited, this might be related to greater difficulties in establishing boundaries and proper classroom management, components of the teaching practice that are partly learned “on the job”. Our findings also show a higher perception of feeling unsafe among teachers who worked mainly in the classroom, a finding suggesting that many situations that may lead to violent behaviors occur in the classroom. Following social–ecological theory, this might be due to the fact that teachers and students share a great deal of time in a confined space together, but also to the fact that victimization and the sense of feeling unsafe are relational consequences of social interactions that occur in these microsystems. Hence, the findings reflect the need to provide in-service teacher training and support, especially for younger teachers working in regular classrooms, particularly regarding classroom management and conflict resolution strategies, since these have been shown to prevent escalating episodes of peer victimization and student-to-teacher victimization (Villarejo-Carballido et al., 2019). Dialogical conflict resolution has been shown to effectively prevent and reduce peer victimization and student-to-teacher victimization in the classroom, increasing teachers’ social support through open communication, collaborative problem-solving, and the enhancement of a defending bystander approach to conflict resolution (Cheon et al., 2023). When young teachers working mainly as regular classroom teachers feel that they can manage their classroom and prevent violent forms of conflict resolution, this probably increases their self-efficacy in terms of feeling capable of and confident in resolving classroom conflicts in the future, which might, in turn, lead to a greater sense of occupational safety.

Our findings highlight how student, parent, and climate variables relate to teachers’ sense of safety in school. Understanding how these variables contribute to feelings of safety among teachers can provide a deeper theoretical understanding of school violence from a socioecological perspective (Astor & Benbenishty, 2019). Teachers’ sense of being safe or unsafe is rooted in layered social, relational, and interpersonally interdependent contexts. In this study, when the school-level teacher, student, and parent reports of school violence were included (model 3), the overarching hypothesis that it is the socioeconomic level of the school (i.e., poor schools) which explains teachers’ feeling unsafe at school (Model 1) lost its explanatory strength (see Models 2, 3, and 4), suggesting that it is more than just SES that explains why teachers feel unsafe. In fact, the significantly higher between-school explained variance as compared to the within-school explained variance (60% versus 14%, respectively) suggests that context matters. Despite this, our findings show that 92% of the variance is explained by within-school differences, that is, due to teacher’s individual and job characteristics, as well as by their perceptions of collegial relationships, student–teacher relationships, and school violence.

Similarly, this study found that teachers’ feeling unsafe at school was associated with their assessments of collegial relationships and teacher–student relationships. Teachers who felt that they got along well with their colleagues and students felt safer in school, compared to those who reported less positive relationships with colleagues and students. These findings are similar to Astor et al.’s (2024) and McMahon et al.’s (2024b) studies in the United States, suggesting that feeling unsafe is related to collegial relationships and student–teacher relationships in other cultural contexts. They also theoretically show that teachers and possibly students (Benbenishty & Astor, 2005) are strongly influenced by their observations of others’ behaviors in school rather than only their personal experiences of victimization. Therefore, our findings reflect the need to foster whole-school approaches to promoting a positive school climate among all members of the school community in different cultural contexts (Capp et al., 2021; López et al., 2025a). In the context of this large-scale sample of lower-SES Chilean schools, who usually have a higher proportion not only of low-SES students but also of students with disabilities and migrant students, and are many times located in unsafe neighborhoods, these findings are highly relevant since they suggest that key interventions for retaining teachers in these school contexts are through strengthening collegial relations and teacher–student relationships, a systemic approach that, in Latin America, is known as *convivencia escolar* or the process of the whole school community learning to live together (Fierro-Evans & Carbajal-Padilla, 2019). Improving *convivencia escolar* protects teachers from feeling the fear of being attacked in school and from feeling that their job is dangerous, a key dimension of occupational safety.

School-related factors related to feeling unsafe at school were the school SES and school size. A lower school SES was linked with an increased sense of feeling unsafe among teachers. Teachers who reported positive relationships with colleagues and students felt safer at school than those with less favorable interactions. This is consistent with prior research in Chile showing that smaller schools foster a more positive climate and lower peer victimization (López et al., 2025b). Following Astor and Benbenishty’s (2019) socioecological theory, this may be possibly due to more frequent teacher–student interactions, which are key to student engagement (April et al., 2023; Cerveny et al., 2022).

The results regarding the impact of the school SES could be explained by how it was measured in this study—as the proportion of students classified as vulnerable by JUNAEB. This means that it is also a measure of the level of support needed by schools, given that the vulnerability index reflected a higher proportion of students living in contexts of poverty. Schools with higher diversity and support needs could have a lower sense of safety. This suggests the need for public policies that provide support to schools with designing and implementing suitable interventions for these students. The variance at the school level, as shown in the variance components, was lower compared to the variance at level 1. The low-level variance means that the level 2 units (e.g., schools) are relatively similar to each other, and the differences in the outcome were more pronounced within schools (Snijders & Bosker, 2012). The similarity between schools could be explained by the homogenic characteristics of the schools in the study—i.e., schools with public funding and with a high proportion of vulnerable students, which, according to the prioritization of JUNAEB, makes them eligible for the SFL program,

One of the limitations of this study is its cross-sectional design; consequently, the relationships between study variables were correlational, and it was not possible to identify causal effects. Although the sample was large, it was not representative of the whole teacher population and, instead, reflected teachers working at lower-SES schools in Chile. Hence, the results are only representative of this subgroup. Future studies should explore these questions and findings with longitudinal samples. Another limitation was the use of the category “other sex”; future studies should seek to more clearly identify teachers’ sexual orientation and gender identities.

Our findings contribute to the knowledge regarding the importance of teaching experience, sex, and working or not in the regular classroom in understanding teachers’ feeling unsafe in schools serving families from lower-SES backgrounds. Context matters for teachers working in lower-SES Chilean schools. Perceptions of school climate and school violence are not independent from students’ and parents’ perceptions but rather partly shaped by both students and parents. Teachers in lower-SES school contexts with a large number of students were more likely to feel less safe. Future research should consider these school-related variables in other parts of the world. They should consider including other variables related to the wider socioecological layer of crime and safety in the community and neighborhoods.

This study examined only low-SES elementary schools. Future studies should include representative samples of teachers from diverse school contexts. Finally, this study highlights the need for culturally comparative studies on individual, school-level, and community-level factors that contribute to teachers’ perceptions of feeling unsafe at school, considering the perspectives of not only teachers but also students and parents.

## Figures and Tables

**Table 1:** Descriptive statistics for study variables for the whole sample.

Variables	M or %	SD	Min	Max
Individual-level variables (*N* = 9639)				
Feeling unsafe at school	0.97	0.85	0	3
Female	72.5			
Male	26.6			
Other sex	0.8			
Aged 18–30	13.5			
Aged 31–45	53.7			
Aged 46 or older	32.8			
Years of experience	13.51	9.82	0	60
Main job outside of classroom	20.8			
Perceived violence	2.68	1.03	1	5
Positive collegial relationships	2.63	0.58	0	3
Positive teacher–student relationships	2.51	0.52	0	3
School-level variables (*N* = 2116)				
Vulnerability index	0.87	0.09	0.42	1.00
Enrollment	346.44	237.02	40	1254
Positive collegial relationships (teachers)	2.52	0.23	1	3
Positive teacher–student relationships (teachers)	2.51	0.22	1.5	3
Perceived violence in school (teachers)	2.68	0.65	1	5
Peer victimization (students)	0.22	0.07	0	2
Student-to-teacher physical victimization	0.05	0.04	0	2
Teacher-to-student victimization	0.08	0.05	0	2
Teachers’ social support	2.18	0.18	0	3
Teacher-to-parent victimization	0.06	0.12	0	2
Positive school–parent relationship	2.08	0.22	0	3

**Table 2:** Summary of multilevel linear regression analysis examining factors associated with teachers’ feeling unsafe at school (*N* = 9625 teachers from 2116 schools).

	Null Model		Model 1		Model 2		Model 3		Model 4	
	*b*	*SE*	*b*	*SE*	*b*	*SE*	*B*	*SE*	*b*	*SE*
Constant	0.938 ***	0.014	−0.293 ***	0.159	−0.139	0.158	0.482	0.250	0.402	0.251
Individual-level variables										
Sex (Ref: female)										
Male					−0.048 *	0.019	−0.043 *	0.018	−0.033	0.017
Other					0.439 ***	0.089	0.405 ***	0.089	0.352 ***	0.083
Years of experience					−0.005 ***	0.001	−0.005 ***	0.001	−0.003 ***	0.001
Main job outside classroom (yes)					−0.171 ***	0.021	−0.180 ***	0.020	−0.149 ***	0.019
Positive collegial relationships (teachers)									−0.104 ***	0.015
Positive teacher–student relationships (teachers)									−0.104 ***	0.017
Perceived violence in school (teachers)									0.275 ***	0.009
School-level variables										
School poverty (IVE)			1.153 ***	0.170	1.112 ***	0.168	0.566 ***	0.138	0.580 ***	0.139
School enrollment			0.001 ***	0.001	0.001 ***	0.001	0.001 *	0.001	0.001 *	0.001
Positive collegial relationships (mean teachers’ perception)							−0.186 ***	0.049	−0.081	0.051
Positive teacher–student relationships (mean teachers’ perception)							−0.238 ***	0.057	−0.081	0.059
Perceived violence in school (mean teachers’ perception)							0.356 ***	0.020	0.081 ***	0.022
Peer victimization (mean students’ reports)							−0.198	0.120	−0.182	0.195
Student-to-teacher victimization (mean students’ reports)							−0.303	0.428	−0.287	0.427
Teacher-to-student victimization (mean students’ reports)							0.577	0.322	0.536	0.320
Teachers’ social support (mean students’ perception)							0.059	0.069	0.066	0.068
Teacher-to-parent victimization (mean parents’ reports)							0.016	0.090	0.014	0.090
Positive school–parent relationship (mean parents’ perception)							0.033	0.055	0.09	0.055
Model fit										
Log likelihood	−11,754.788		−11,701.032		−11,623.15		−11,408.011		−10,859.24	
AIC	23,515.58		23,412.06		23,264.29		22,852.02		21,734.01	
BIC	23,537.1		23,447.93		23,328.85		22,981.15		21,884.66	
Variance components										
Teacher	0.604		0.604		0.594		0.594		0.523	
School	0.128		0.107		0.104		0.039		0.047	
% Level 1 explained variance			0.0		1.7		1.7		13.4	
% Level 2 explained variance			16.4		18.8		69.53		63.3	

Note. *SE* = standard error. Level 1 and Level 2 explained variance is the percentage compared to the null model. * *p* < 0.05. *** *p* < 0.001.

## Data Availability

The data that support the findings of this study are available from the first author.
